# Complete Genome Sequences of Isolates of *Enterococcus faecium* Sequence Type 117, a Globally Disseminated Multidrug-Resistant Clone

**DOI:** 10.1128/genomeA.01553-16

**Published:** 2017-03-30

**Authors:** Ana P. Tedim, Val F. Lanza, Marina Manrique, Eduardo Pareja, Patricia Ruiz-Garbajosa, Rafael Cantón, Fernando Baquero, Teresa M. Coque, Raquel Tobes

**Affiliations:** a Microbiology Department, Hospital Universitario Ramón y Cajal, Instituto Ramón y Cajal de Investigación Sanitaria (IRYCIS), Madrid, Spain; b Centros de Investigación Biomédica en Red de Epidemiología y Salud Pública (CIBER-ESP), Madrid, Spain; c ERA7 Bioinformatics, Madrid, Spain; d Red Española de Investigación de Patologías infecciosas (REIPI), Madrid, Spain

## Abstract

The emergence of nosocomial infections by multidrug-resistant sequence type 117 (ST117) *Enterococcus faecium* has been reported in several European countries. ST117 has been detected in Spanish hospitals as one of the main causes of bloodstream infections. We analyzed genome variations of ST117 strains isolated in Madrid and describe the first ST117 closed genome sequences.

## GENOME ANNOUNCEMENT

A recent worldwide increase in multidrug-resistant (MDR) *Enterococcus faecium* strains that cause infections in hospitals has been associated with the abrupt emergence of *E. faecium* populations belonging to the lineage 78. Among this lineage, isolates identified as sequence type 117 (ST117) are predominantly recovered in many European health institutions (1–6). In Spain, ST117 *E. faecium* isolates are increasingly identified from clinical isolates and have been identified as causing bloodstream infections (BSIs) since 2006 (7).

We report here the sequences of five ST117 *E. faecium* isolates belonging to an endemic clone causing bacteremia and frequently colonizing hospitalized patients in our hospital, mostly at the gastroenterology (30.1%) and hematology (16.4%) wards (5, 7). All isolates were resistant to ampicillin, erythromycin, ciprofloxacin, and levofloxacin and, eventually, resistant to high levels of streptomycin, gentamicin, and tetracycline. Comparative genomics between these five ST117 genome sequences and others available at the GenBank database will help elucidate possible factors that might have contributed to the emergence of particular clones.

Five ST117 isolates associated with bloodstream (*n* = 4) and urinary tract (*n* = 1) infections were selected for this study based on their antibiotic susceptibilities, virulence/colonization traits, and origin (community and hospital) (5, 7). DNA extraction was performed with the Wizard genomic DNA purification kit (Promega, Madison, WI). DNA concentration was measured with a NanoDrop 2000 (Thermo Scientific) and Qubit 2.0 fluorometer (Life Technologies, Inc.). All strains were sequenced using Illumina (2 × 101-bp, 900× coverage), with one of them also being sequenced by PacBio (800× coverage) in order to close the first genome of this pandemic lineage. The Illumina reads and Illumina plus PacBio reads were assembled using SPAdes 3.5.0 (8) and RS_HGAP_Assembly.2 (9). Genome annotation was performed using PGAAP from NCBI (10) at the GenBank submission step.

The genome of the *E. faecium* ST117 E1 strain has a circular chromosome with 2,925,525 bp and a G+C content of 38.0%, 2,862 coding sequences (CDS), 71 tRNAs, and six rRNA operons. E1 strain harbors four circular plasmids ranging from 3,296 bp to 230,049 bp: pE1_230 (230,049 bp), pE1_29 (29,012 bp), pE1_13 (13,112 bp), and pE1_3 (3,199 bp).

The PacBio assembly yields a genome of significant larger size than that obtained by using Illumina. The “genome gain” mainly corresponded to transposases, insertion sequences, and RNA operon copies that were probably collapsed during the Illumina assembly process. Thus, our results reflect the suitability of PacBio for fully characterizing plasmids and virulence islands of enterococci, which are chimeric elements that are impossible to elucidate by using other next-generation sequencing (NGS) approaches (6, 11). The more closely related strains were ST117 strains isolated in our hospital (<12 SNPs). The overall characteristics of the E1 genome were similar to those of another closed genome of the ST78 lineage (strain AUS0085) (12). Further studies of comparative genomics studies are needed in order to understand the emergence of the ST78 lineage in the hospital setting.

### Accession number(s).

The GenBank genome accession numbers are CP018065 to CP018069 (E1), MPZY00000000 (E2), MPZZ00000000 (E3), MQAA00000000 (E4), MQAB00000000 (E5), and MQAC00000000 (E6).

## References

[1] Werner G, Coque TM, Hammerum AM, Hope R, Hryniewicz W, Johnson A, Klare I, Kristinsson KG, Leclercq R, Lester CH, Lillie M, Novais C, Olsson-Liljequist B, Peixe LV, Sadowy E, Simonsen GS, Top J, Vuopio-Varkila J, Willems RJ, Witte W, Woodford N 2008 Emergence and spread of vancomycin resistance among enterococci in Europe. Euro Surveill 13:pii=19046 http://www.eurosurveillance.org/ViewArticle.aspx?ArticleId=19046. 19021959

[2] Arias CA, Murray BE 2012 The rise of the *Enterococcus*: beyond vancomycin resistance. Nat Rev Microbiol 10:266–278. doi:10.1038/nrmicro2761. 22421879 PMC3621121

[3] Sundsfjord A, Willems R 2010 *Enterococcus* research: recent developments and clinical challenges. Clin Microbiol Infect 16:525–526. doi:10.1111/j.1469-0691.2010.03215.x. 20569262

[4] Willems RJL, Top J, van Schaik W, Leavis H, Bonten M, Sirén J, Hanage WP, Corander J 2012 Restricted gene flow among hospital subpopulations of *Enterococcus faecium*. mBio 3:e00151-12. doi:10.1128/mBio.00151-12. 22807567 PMC3413404

[5] Tedim AP, Ruiz-Garbajosa P, Corander J, Rodríguez CM, Cantón R, Willems RJ, Baquero F, Coque TM 2015 Population biology of intestinal *Enterococcus* isolates from hospitalized and nonhospitalized individuals in different age groups. Appl Environ Microbiol 81:1820–1831. doi:10.1128/AEM.03661-14. 25548052 PMC4325138

[6] Freitas AR, Tedim AP, Francia MV, Jensen LB, Novais C, Peixe L, Sánchez-Valenzuela A, Sundsfjord A, Hegstad K, Werner G, Sadowy E, Hammerum AM, Garcia-Migura L, Willems RJ, Baquero F, Coque TM 2016 Multilevel population genetic analysis of *vanA* and *vanB Enterococcus faecium* causing nosocomial outbreaks in 27 countries (1986–2012). J Antimicrob Chemother 71:3351–3366. doi:10.1093/jac/dkw312. 27530756

[7] Tedim AP, Ruíz-Garbajosa P, Rodríguez MC, Rodríguez-Baños M, Lanza VF, Derdoy L, Cárdenas Zurita G, Loza E, Cantón R, Baquero F, Coque TM 2017 Long-term clonal dynamics of *Enterococcus faecium* strains causing bloodstream infections (1995–2015) in Spain. J Antimicrob Chemother 72:48–55. doi:10.1093/jac/dkw366. 27655856

[8] Bankevich A, Nurk S, Antipov D, Gurevich AA, Dvorkin M, Kulikov AS, Lesin VM, Nikolenko SI, Pham S, Prjibelski AD, Pyshkin AV, Sirotkin AV, Vyahhi N, Tesler G, Alekseyev MA, Pevzner PA 2012 SPAdes: a new genome assembly algorithm and its applications to single-cell sequencing. J Comput Biol 19:455–477. doi:10.1089/cmb.2012.0021. 22506599 PMC3342519

[9] Chin CS, Alexander DH, Marks P, Klammer AA, Drake J, Heiner C, Clum A, Copeland A, Huddleston J, Eichler EE, Turner SW, Korlach J 2013 Nonhybrid, finished microbial genome assemblies from long-read SMRT sequencing data. Nat Methods 10:563–569. doi:10.1038/nmeth.2474. 23644548

[10] Angiuoli SV, Gussman A, Klimke W, Cochrane G, Field D, Garrity GM, Kodira CD, Kyrpides N, Madupu R, Markowitz V, Tatusova T, Thomson N, White O 2008 Toward an online repository of Standard Operating Procedures (SOPs) for (Meta)genomic annotation. OMICS 12:137–141. doi:10.1089/omi.2008.0017. 18416670 PMC3196215

[11] Novais C, Tedim AP, Lanza VF, Freitas AR, Silveira E, Escada R, Roberts AP, Al-Haroni M, Baquero F, Peixe L, Coque TM 2016 Co-diversification of *Enterococcus faecium* core genomes and PBP5: evidences of *pbp5* horizontal transfer. Front Microbiol 7:1581. doi:10.3389/fmicb.2016.01581. 27766095 PMC5053079

[12] Lam MMC, Seemann T, Tobias NJ, Chen H, Haring V, Moore RJ, Ballard S, Grayson LM, Johnson PDR, Howden BP, Stinear TP 2013 Comparative analysis of the complete genome of an epidemic hospital sequence type 203 clone of vancomycin-resistant *Enterococcus faecium*. BMC Genomics 14:595. doi:10.1186/1471-2164-14-595. 24004955 PMC3846456

